# Cardiovascular disease medication health literacy among Indigenous peoples: design and protocol of an intervention trial in Indigenous primary care services

**DOI:** 10.1186/1471-2458-14-714

**Published:** 2014-07-12

**Authors:** Sue Crengle, Janet Smylie, Margaret Kelaher, Michelle Lambert, Susan Reid, Joanne Luke, Ian Anderson, Jennie Harré Hindmarsh, Matire Harwood

**Affiliations:** 1 National Institute of Health Innovation, School of Population Health, University of Auckland, Auckland, New Zealand; 2 Centre For Research on Inner City Health, Li Ka Shing Ka Shing Knowledge Institute, Saint Michael’s Hospital, Toronto and Dalla Lana School of Public Health, University of Toronto, Toronto, Canada; 3 Centre for Health Policy, Programs & Economics, Melbourne School of Population and Global Health, University of Melbourne, Melbourne, Australia; 4 Workbase Education Trust, Ponsonby, Auckland, New Zealand; 5 The Victorian Aboriginal Health Service, Fitzroy, Australia; 6 Murrup Barak, Melbourne Institute for Indigenous Development, University of Melbourne, Melbourne, Australia; 7 Ngati Porou Hauora Charitable Trust, Gisborne, New Zealand; 8 Te Kupenga Hauora Maori, Faculty of Medical and Health Sciences, University of Auckland, Auckland, New Zealand

**Keywords:** Indigenous health, Health literacy, Cardiovascular diseases, Medication knowledge, Non-randomized study design, Clinical trial

## Abstract

**Background:**

Cardiovascular diseases (CVD) are leading causes of mortality and morbidity among Indigenous people in New Zealand, Australia and Canada and are a major driver of the inequities in life expectancy between Indigenous and non-Indigenous people in these countries. Evidence-based pharmaceutical management of CVD can significantly reduce mortality and morbidity for persons diagnosed with CVD or for those at intermediate or high risk of CVD. Health literacy has been identified as a major barrier in the communication and implementation of appropriate pharmaceutical management plans for CVD. Addressing health literacy is particularly relevant in Indigenous populations where there are unique health and adult literacy challenges.

**Methods/design:**

This study will examine the effect of a customized, structured CVD medication programme, delivered by health professionals, on the health literacy of Indigenous people with, or at risk, of CVD. Primary outcomes are patient’s knowledge about CVD medications; secondary outcomes examine changes in health literacy skills and practices. The study will employ a multi-site pre-post design with multiple measurement points to assess intervention efficacy. Participants will be recruited from four Indigenous primary care services in Australia, Canada and New Zealand. Three educational sessions will be delivered over four weeks. A tablet application will support the education sessions and produce a customized pill card for each participant. Participants will be provided with written information about CVD medications. Medication knowledge scores, and specific health literacy skills and practices will be assessed before and after the three sessions. Statistical analyses will identify significant changes in outcomes over each session, and from the pre-session one to post-session three time points.

**Discussion:**

This study will make an important contribution to understanding the effect of a structured primary care-based intervention on CVD health literacy in Indigenous populations. The study also illustrates the incorporation of Indigenous health research principles and processes in clinical trials and provides insights that may be useful in other contexts.

**Trial registration:**

Australian and New Zealand Clinical Trials Register (ACTRN12612001309875; date of registration 18/12/2012).

## Background

Cardiovascular diseases are major causes of mortality and hospitalisation for adult Indigenous peoples. Significant inequalities in CVD morbidity, mortality and the prevalence of cardiovascular disease risk factors between Indigenous and non-Indigenous populations have been documented in Australia [1] Canada [2], and New Zealand [3,4]. Explicit evidence-based guidelines for the management of CV risk factors and CVD have been available for many years. Healthy nutrition, exercise, smoking cessation, and medications are the key strategies of primary and secondary prevention. Initiatives such as explicit evidence-based CVD management guidelines [5], health provider education [6] and computerised decision support tools [7-11] have been implemented to assist health professionals and health services to provide high quality CVD care. However, CVD are chronic diseases and as such, the patient and their family are the primary ‘managers’ of these diseases. Self-management is central to effective CVD management [12]. A complex array of skills, knowledge and psycho-social factors influence a person’s ability to be an effective self-manager of chronic conditions [13]. Knowledge about CVD risk factors and medications is essential to self-management. Published literature on patients’ knowledge focuses on CVD risk factors and understanding of CVD risk assessment rather than knowledge and understanding of medications and their use [14-18]. Available evidence suggests that intermittent and non-adherence with medications is common and is associated with worse outcomes, including poorer control of risk factors, increased hospitalisations, morbidity and mortality [19-22].

Health literacy, defined as “the ability to access, understand and act on information for health” [23], is an important contributor to knowledge, self-management, and health outcomes generally [24-30] and for CVD [31]. The Institute of Medicine has reported that differences in health literacy alone account for 25–30 per cent of ethnic differences in health outcomes [32]. International surveys have found that the majority of the population in Australia, Canada and New Zealand (NZ) have inadequate levels of health literacy to effectively engage in health care. In NZ, Māori have higher health literacy needs than non-Māori [33,34]. While rigorous population based Indigenous data are lacking in Australia and Canada, the situation for Indigenous peoples in both countries is likely to be similar to New Zealand given the known inequities in health and education [35].

The intervention used in this trial was designed during a developmental phase. This included in-depth interviews with community members who were taking CVD prevention medications to ascertain their knowledge about their medications, what they would like to know about these medications, and how they would like to be provided with this information. There were significant knowledge gaps at all four locations and, while there was some locality variation, the knowledge gaps and expressed information needs were remarkably similar across sites. The data from these interviews were used to develop the intervention. Interviews with health professionals were also undertaken and explored their awareness and understanding of health literacy and how they addressed health literacy in their practice. Environmental scans have assessed the baseline health literacy demands the health service environment places on its clients. Data from health professional interviews and environmental scans will allow assessment of whether changes in health professionals’ awareness, knowledge and practices and changes in the health literacy environment may contribute to the results of the intervention trial.

### Study aims

The aim of this study is to test the effect of a customized, structured CVD medication programme, delivered by health professionals, on the related health literacy of Indigenous people with, or at risk, of CVD. Primary outcomes are patient’s knowledge about CVD medications. Secondary outcomes examine changes in health literacy skills and practices. The programme will be implemented in Indigenous primary health care services in Australia (one service), Canada (one service with two sites), and NZ (two services). The intervention will be delivered by nurses in NZ and Canada, and a researcher/health educator in Australia.

## Methods and design

The intervention trial utilizes a single arm pre-post design with multiple measurement points. Two factors related to the size of the local Indigenous communities preclude the use of randomised, controlled trial design. Firstly, there is a high likelihood of contamination between control and intervention groups in the Indigenous communities where the trial will be located. Secondly, the use of a control group is not feasible in these communities because the eligible population is relatively small and, in order to have sufficient participants receiving the intervention, all eligible people will have to receive it. The uniqueness of local Indigenous communities and their health services precluded the use of quasi-experimental design with matched primary care service controls located elsewhere.

### Indigenous community engagement in the research process

In keeping with ethical requirements and evidence based best practices for Indigenous research [36-39], as well as some site’s own research policies [40,41], the participating Indigenous communities have been involved in the development of the project from its inception.

The principal investigators in all three countries are Indigenous peoples and all the Indigenous researchers have pre-existing relationships with the participating Indigenous health services where the research will be based. These services were involved in all aspects of project development including decisions to submit a grant application, development of the application, study design, and the implementation of phase one of the project. Key staff from the health services are project co-investigators, and the intervention will be delivered by Indigenous staff employed by the health services. In all three countries specific policies and processes regarding project governance and implementation; health service provider/community expectations; benefits of the research; ownership, control, and access to research information, and dissemination of project results, including academic publications, have been agreed.

### Ethics

The study design and implementation processes were reviewed and approved by each of the four participating Indigenous health services. The study was approved by the Health and Disabilities Ethics Committees in NZ (MEC/10/061/EXP), the Human Research Ethics Committee (HREC) at the University of Melbourne, Australia and the Saint Michael’s Hospital Research Ethics Board, Toronto, Canada. The study was registered with the Australian and New Zealand Clinical Trials Register on 18th December 2012 (ACTRN12612001309875).

### Study locations

The study will be implemented in four Indigenous health services (five sites) across the three countries. In NZ, one service (Te Hononga o Tamaki me Hoturoa) is located in a large metropolitan city that provides nurse-led services but does not provide primary medical care services. Participants from this service will be drawn from nurse-led programmes for people with long-term conditions and for people recently discharged from hospital with a diagnosis of a CVD event. The second NZ site is located in a remote rural area whose health services are provided by Ngati Porou Hauora, an Indigenous health provider that delivers a range of primary care services including medical services.

The Australian partner is the Victorian Aboriginal Health Service (VAHS). VAHS is an urban community controlled Aboriginal health service that was established in 1973 to address the specific medical needs of local Victorian Aboriginal communities. VAHS now provides a comprehensive range of medical, dental and social services and supports the well-being of the community through contributions to community events and activities.

The Canadian study partner is the Dedwadadehsnye > s Aboriginal Health Access Centre (DAHAC), a provincially funded urban Indigenous health service which provides primary health care, traditional healing and health promotion programs to Indigenous people living in the cities of Hamilton and Brantford, Ontario. Recruitment and intervention will occur at both DAHAC sites, which are 40 km apart and have similarly structured primary health care services.

### Recruitment

Medical records at the sites will be systematically searched to identify potentially eligible patients. These patients will be contacted by a health service staff member who is not directly involved in the project and asked if they would like to know more about the trial. Patients who accept this invitation will then be contacted by the nurse/health educator who will provide information about the study and undertake formal consent procedures with patients that agree to participate. Participants will be able to invite family members to sit in on the sessions; however, data will not be collected from family members.

### Eligibility

Patient participants are eligible for the trial if they are Indigenous adults ≥ 20 years of age and are enrolled with the participating health services. In addition they need to have been diagnosed with angina pectoris, myocardial infarction, transient ischaemic attacks or stroke OR (for Canada and Australia only) were known to be at risk of a CVD event and receiving medication to manage this risk. In Australia these participants were eligible if they were at high risk (≥15 % risk of a CVD event in the next five years) [36]. In Canada participants were eligible if they were assessed as having at least internediate risk (≥15 % risk of an event in the next 10 years) [42,43]. Finally, all participants need to be taking at least two of the following types of medicines: a statin, aspirin, a beta blocker, or an ACE inhibitor. Eligible participants will be excluded from the trial if they have a condition which means they are unable to provide informed consent or they have a cognitive deficit that interferes with their ability to participate in the intervention.

### Power analysis

A power analysis that allows for the conduct of country specific analyses was undertaken. Hill-Briggs [44] found that a literacy adapted diabetes and CVD education intervention among an urban African American population resulted in a change in mean knowledge score from 2.7 ± 1.7 to 4.7 ± 2.0 (maximum score possible = 9) among people with low health literacy. Assuming the proposed intervention will result in small to medium improvements in medication knowledge and health literacy practices, the minimum sample size required at each of the five sites to detect an effect size of 0.4 respectively for mean pre-post test scores with a power (1-β) of 0.8 and alpha of 0.05 is 52. For an effect size of 0.3, 90 patients would be needed. Our recruitment targets are, therefore, 100 participants at each of the two NZ sites, 100 participants in Australia, and 120 at each Canadian site.

### Intervention

The intervention consists of an educational session delivered by health professionals who will have received training in health literacy, and strategies to support knowledge acquisition and the development of health literacy skills using adult education principles. A booklet, designed by the medical and health literacy research team members, will be provided to participants. It includes information about CVD, and medication use in general (including prescription labels, side effects, keeping medicines safely, taking medicines safely, and interactions). The booklet also contains specific information about the four CVD medication classes (statins, aspirin, beta blockers, and ACE inhibitors) including the scientific and brand names of the medications, what the medication does, how to take it, interactions, side effects, contraindications to taking the medicine, what to do if experiencing a serious side effect, how to know if the treatment is working, and lipid and blood pressure treatment targets.

An interactive tablet application has been developed for use during each session. This tablet ensures that the nurse/educator covers the CVD medication information in a structured way and provides interactive opportunities for the participant. The application also prints out a participant specific pill card with images of their medications. A list of three participant identified questions for use next time they see their health professional is also printed. In the context of the trial the application will also ensure that information is provided in a standardised manner across all sites.

The written resource and tablet information were standardized across all three countries, however images and background graphic design features varied with local Indigenous culture and language being used in each country.

Participants will only cover information about the medication classes they are currently taking. That is, if they are currently taking a statin and an ACE inhibitor they will cover these two medication types but will not receive information about aspirin and beta blockers.

The education session will be delivered on three occasions at intervals determined using adult education principles to optimise learning opportunities. The second session is scheduled to occur seven days after the first but can be completed up to two weeks after the scheduled date. The third session is scheduled to occur 28 days after the first session but can be completed up to four weeks after the scheduled date.

The time required to complete each session will vary depending on the number of medications the participant is taking. We anticipate that each session will be no less than 30 minutes and no more than 75 minutes.

Training for research staff will be provided in New Zealand (NZ and Australian research staff) and in Canada. Training sessions with research staff from all three countries will also be undertaken using teleconferences and Skype. Training will be provided in health literacy, principles of adult learning, the delivery of the intervention, and all trial protocols and processes including data collection and quality assurance processes. A detailed training manual will be provided. Training in use of the medication knowledge outcome measures will include the use of standardized scenarios that will be pre-scored by two investigators (SC, SR). Each nurse/educator will score the scenario using the relevant medication knowledge outcomes measures. This process will continue with supplementary training where needed until accuracy and cross-site concordance in scoring is reached. To evaluate scoring practices during the trial this process will be repeated at regular intervals using standardized video scenarios.

### Main study outcomes

The primary study outcome measures will assess changes from baseline to data collection time points for: (1) patient knowledge about the CVD medication classes they are taking; (2) patient knowledge of their treatment targets for lipids and blood pressure; and (3) the use of participant-specified questions with their usual healthcare provider. Secondary outcomes measures will examine changes in patient health literacy skills and practices. Table 1 describes the data collected at the measurement points.

**Table 1:** Summary of trial contacts and data collection

**Time point**		**Measurement**
Enrolment visit.	T0	Consent and enrolment in study. In Canada baseline demographic and clinical information was also collected at this visit
Session one.	T1	Baseline demographic and clinical information (NZ, Australia)
		Pre-session one medication knowledge and health literacy practices
	T2	Post session one medication knowledge and health literacy practices
Session two.	T3	Pre session two medication knowledge and health literacy practices
Seven days after session one.		Health literacy practices between sessions one and two
	T4	Post session two medication knowledge and health literacy practices
Session three.	T5	Pre session three medication knowledge and health literacy practices
28 days after session one.		Health literacy practices between sessions two and three
	T6	Post session three medication knowledge and health literacy practices

### Measurement instruments

Baseline demographic, medical history, medication information, blood pressure and lipid recordings will be collected at the enrolment session or at the start of the first session (T0 or T1 in Table 1). Outcome assessment tools that assess patient knowledge of the scientific and brand name of their medications, class of drug, function of drug, important side effects, special instructions for taking the medicine, and (where relevant) knowledge of lipid and BP treatment targets have been developed for statin, aspirin, beta blockers and ACE inhibitors and will be completed immediately before and after each education session (T1-T6).

Participants’ health literacy skills and practices during each outcome assessment will be assessed (T1-T6). Participants will be asked about their health literacy practices prior to the first session and between each session (T0/T1, T3 and T5). Participants will be asked about their use of their questions for health professionals at the beginning of the second and third sessions (T3 and T5).

### Statistical analyses

Descriptive statistics will describe the baseline characteristics of study participants (age, sex, CVD diagnosis, co-morbidities, medications), baseline blood pressure and lipid measures, and the characteristics of the sessions with respect to location (home/office) and whether or not family members were present. Finally we will describe the baseline health literacy skills and practices of participants including how and from where/whom they acquire information CVD medications.

To assess the effect of the intervention on patient knowledge about their medication we will examine the mean knowledge scores for each of the four CVD medication groups over time. We will test changes in mean scores from pre session one (T1) to post session three (T6). Baseline characteristics of participants who enrolled but did not complete the study will be compared to participants who finished the intervention to examine whether drop out was determined by specific characteristics or not.

## Discussion

In all three countries data obtained in the developmental phase showed significant gaps in patients’ cardiovascular medication knowledge that are likely to interfere with safe and effective medication use. Improving health literacy about CVD medications is one of a range of strategies necessary to reduce the inequitable burden of cardiovascular disease experienced by Indigenous populations. This study will determine if health professional delivered, health literacy oriented educational sessions delivered in the primary care setting enhances medication knowledge and health literacy practices within Indigenous communities in Australia, Canada and New Zealand. If successful the intervention may also be useful for a wider range of medications associated with other long-term conditions and in non-Indigenous contexts.

The study is one of the first international clinical trials conducted solely among Indigenous populations. Building on previously published work about Indigenous involvement in clinical trials [45] the protocol illustrates the application of Indigenous health research principles and protocols [46], such as Indigenous involvement across all roles and throughout the entire project, to clinical trials. Implementation of these principles and processes requires appropriate relationships and communication with the health services, their staff and the study participants. The use of outcomes measures that assess the development of knowledge, skills and practices is informed by Indigenous preferences for strength-based rather than deficit focused research. This strength-based approach is also consistent with Nutbeam’s [30] approach to health literacy.

The study design also illustrates one approach to implementing clinical trials in distinctive local contexts. In Canada, networks of friends and family have been identified by members of our research team as the most important source of health information in diverse local Indigenous communities [47]. Furthermore, the relatively small population sizes of these communities means that, in order to have a sufficient sample to assess the effect of the intervention, it will have to be delivered to all eligible people in the health services. The study design needs to accommodate both these challenges, making a single arm, pre-post design with case studies of the surrounding environments for factors that may influence the effect of the trial, the most suitable design. Similar challenges may be encountered in other studies that are located in relatively small, local populations.

A key audience for our study results will be the Indigenous health service providers who are responsible for providing care to the patients in the study. We will work with these health service provider partners to interpret the study findings and identify recommendations for their practice. We will also share our study findings more broadly with Indigenous and non-Indigenous health policy makers, practitioners, and researchers.

## Competing interests

The authors declare that they have no competing interests.

## Authors’ contributions

SC conceived of the study, led the design, wrote the protocol, led the NZ research team, co-wrote the manuscript. JS contributed to study design, led the Canadian team, co-wrote the first draft and provided later comment. MK participated in the design, led their country specific research teams and collaborated in drafting the manuscript. ML contributed to writing the protocol, participated in study design, was responsible for international coordination of the study, helped draft the manuscript and prepared it for submission. SR, IA, JL, JH and MH participated in study design and helped to draft the manuscript. All authors read and approved the final manuscript.

## Pre-publication history

The pre-publication history for this paper can be accessed here:

http://www.biomedcentral.com/1471-2458/14/714/prepub
